# Natural Language Processing to Classify Caregiver Strategies Supporting Participation Among Children and Youth with Craniofacial Microsomia and Other Childhood-Onset Disabilities

**DOI:** 10.1007/s41666-023-00149-y

**Published:** 2023-09-18

**Authors:** Vera C. Kaelin, Andrew D. Boyd, Martha M. Werler, Natalie Parde, Mary A. Khetani

**Affiliations:** 1 Department of Occupational Therapy, University of Illinois Chicago, 1919 West Taylor Street, Room 316A, Chicago, IL 60612 − 7250 USA; 2 Department of Computer Science, University of Illinois Chicago, 851 South Morgan Street, Room 1132, Chicago, IL 60607-7042 USA; 3 Children’s Participation in Environment Research Lab, University of Illinois Chicago, Chicago, IL USA; 4 Biomedical and Health Information Sciences, University of Illinois Chicago, Chicago, IL USA; 5 Epidemiology, Boston University, Boston, MA USA; 6 Natural Language Processing Laboratory, University of Illinois Chicago, Chicago, IL USA; 7 CanChild Centre for Childhood Disability Research, McMaster University, Hamilton, ON Canada

**Keywords:** Pediatric rehabilitation, Artificial intelligence, Activities, Preferences, Sense of self, Environment

## Abstract

Customizing participation-focused pediatric rehabilitation interventions is an important but also complex and potentially resource intensive process, which may benefit from automated and simplified steps. This research aimed at applying natural language processing to develop and identify a best performing predictive model that classifies caregiver strategies into participation-related constructs, while filtering out non-strategies. We created a dataset including 1,576 caregiver strategies obtained from 236 families of children and youth (11–17 years) with craniofacial microsomia or other childhood-onset disabilities. These strategies were annotated to four participation-related constructs and a non-strategy class. We experimented with manually created features (i.e., speech and dependency tags, predefined likely sets of words, dense lexicon features (i.e., Unified Medical Language System (UMLS) concepts)) and three classical methods (i.e., logistic regression, naïve Bayes, support vector machines (SVM)). We tested a series of binary and multinomial classification tasks applying 10-fold cross-validation on the training set (80%) to test the best performing model on the held-out test set (20%). SVM using term frequency-inverse document frequency (TF-IDF) was the best performing model for all four classification tasks, with accuracy ranging from 78.10 to 94.92% and a macro-averaged F1-score ranging from 0.58 to 0.83. Manually created features only increased model performance when filtering out non-strategies. Results suggest pipelined classification tasks (i.e., filtering out non-strategies; classification into intrinsic and extrinsic strategies; classification into participation-related constructs) for implementation into participation-focused pediatric rehabilitation interventions like Participation and Environment Measure Plus (PEM+) among caregivers who complete the Participation and Environment Measure for Children and Youth (PEM-CY).

**Supplementary Information:**

The online version contains supplementary material available at 10.1007/s41666-023-00149-y.

## Introduction

Participation in meaningful activities is a key rehabilitation outcome [1] and important to child and youth inclusion and skill development [2, 3]. To support participation of children and youth with disabilities, a literature review by Adair and colleagues [4] emphasizes the importance of customizing intervention to the individual needs of families and their children and youth. Coaching children and families to generate customized participation-focused strategies for a specific participation challenge (e.g., “re-structuring the physical space in which the activity occurs” [5](p.5) or “using visual cues” [6](p.193)) is a main ingredient of two participation-focused interventions with evidence supporting their effectiveness to improve participation (i.e., the Pathways and Resources for Engagement and Participation (PREP) and the Occupational Performance Coaching (OPC)) [3, 7–9]. There is growing evidence of participation-focused caregiver strategies as drivers of child and youth participation; one revealing a direct effect of participation-focused caregiver strategies on home participation when combined with pediatric rehabilitation services for critically ill children [10] and one revealing an indirect effect of participation-focused caregiver strategies on the relationship between environmental support and school participation frequency for children and youth with craniofacial microsomia and other childhood-onset disabilities. [11]

Although promising, customizing participation-focused interventions for children and youth is a complex and potentially resource intensive process [12]. Technology might support rehabilitation practitioners to feasibly customize participation-focused interventions by automating and simplifying steps and, thus, support to deliver family-centered interventions to service eligible children, youth and families [13, 14]. However, two scoping reviews on the use of AI in participation-focused pediatric re/habilitation indicated a lack of AI-based tools that are customized to individual needs [15, 16]. The Participation and Environment Measure Plus (PEM+) is being designed to pair with a Participation and Environment Measures (PEM) and is a promising technology-based and remotely delivered intervention for engaging families in decisions about service design [17–19]. PEM + currently provides an online option for guiding caregivers to prioritize their participation goals and use a strategy exchange feature to identify participation-focused strategies for goal attainment. There is preliminary evidence of PEM + usability, acceptability, feasibility, and preliminary effects on caregiver confidence [17, 18]. However, PEM + acceptability results highlight the need to further optimize its strategy exchange feature to provide for a more customized user experience when searching for strategies to support goal attainment [17, 18]. Specifically, caregivers need to be able to more easily search for strategies to support their child’s participation in a priority activity. Caregiver strategies from the Young Children’s PEM and included in the PEM + strategy exchange feature are manually classified into four evidence-based categories of participation-related constructs (i.e., child’s environment/context, sense of self, preferences, activity competencies) that are drivers of participation [20], while filtering out caregiver entries that do not classify as strategies (e.g., “none”) [21]. Since manual coding of these narrative strategies data is not sustainable, there is need to explore use of natural language processing (NLP) to automate the customization of applications like the PEM + strategy exchange feature [22].

The purpose of this study is to apply NLP to develop and identify a best performing predictive model that classifies PEM caregiver strategies into participation-related constructs consisting of extrinsic factors (i.e., environment/context) and intrinsic factors (i.e., sense of self, preferences, activity competence [20]), while filtering out non-strategies.

This study makes the following contributions:

1. Create predictive models to classify participation-focused caregiver strategies, as a first step to automate customization of participation-focused pediatric rehabilitation interventions.
2. Provide preliminary evidence on meaningful features to include in predictive models for classifying participation-focused caregiver strategies.

Study results could have immediate impact in advancing the intentional use of AI in participation-focused pediatric re/habilitation. The most proximal points of impact are in reinforcing decisions to pursue NLP for customizing the existing PEM + application and in guiding decisions about further adapting and testing it for use by caregivers of children and youth who complete the PEM for Children and Youth (PEM-CY) [23].

## Related Work

Despite rapid increase in the use of artificial intelligence in pediatric rehabilitation, classification of caregiver strategies has been limited [15, 16]. NLP research on classifying functional outcomes such as activity competence or participation may be most closely related to the classification of caregiver strategies to support child and youth participation.

Kukafka and colleagues (2006) [24] experimented with automated coding of inpatient rehabilitation discharge summaries to five codes within the International Classification of Functioning, Disability and Health (ICF) (i.e., b117 intellectual functions, d420 transferring oneself, d530 toileting, d550 eating, d5400 putting on clothes) using the Medical Language Extraction and Encoding system (MedLEE) [25], a medical language processing program that uses a rule-based approach. Receiver operating characteristic (ROC) curves of classifier performance ranged from 0.52 to 0.82.

In 2021, three studies were published on the use of different NLP approaches to classify narrative data to ICF codes pertaining to the “Activity and Participation” domain within the ICF [26–28]. Thieu and colleagues (2021) [26] linked 400 physical therapy records to ICF codes within the Mobility chapter. Narrative data were tagged as ‘mobility activity report’, which were further annotated as ‘Actions’ (e.g., walking), ‘Assistance’ (e.g., with cane), and ‘Quantification’ (e.g., 300ft). To classify the data, Thieu and colleagues (2021) [26] used popular classification models for sequential data (i.e., Conditional Random Field (CRF) [29], Recurrent Neural Networks (RNN) [30], and Bidirectional Encoder Representation from Transformers (BERT) [31]). The Ensemble method, which combined outputs of multiple classifiers, performed best (F1 = 0.85).

Newman-Griffis and colleagues (2021) [28] used this same annotated dataset to examine different types of word representations (unigram features, static word embeddings, and contextualized word embeddings) and classification approaches. Static word embeddings (i.e., word2vec) were trained on GoogleNews, MIMIC-III dataset, electronic health record (EHR) notes, and additional physical therapy (PT) and occupational therapy (OT) records. For contextualized embeddings, benchmark pre-trained BERT models (i.e., BERT-base [31], BioBERT [32], clinicalBERT [33]) were used. To classify the narrative data, authors applied support vector machines (SVM), Deep Neural Network (DNN), *k*-Nearest Neighbors (KNN) and candidate selection approaches with and without an ‘Action oracle’ approach (i.e., tagged ‘Action’ within a tagged ‘Mobility activity’ is provided a priori to the coding model). The best performing model applied SVM using ‘Action oracle’ and static embeddings, which were pre-trained on PT/OT records (F1 = 0.84).

Newman-Griffis and colleagues (2021) [27] have also linked content from claims data for federal disability benefits to ‘Mobility’ and ‘Self-care/Domestic Life’ chapters within the ICF ‘Activity and Participation’ domain. Authors created static word embeddings using FastText [34] and pre-trained them on MIMIC-III, EHR records, and Social Security Administration (SSA) documentation. For contextualized word embeddings clinicalBERT [33] was used. Authors applied a SVM classifier and DNN for candidate selection, both with and without the ‘Action oracle’ approach, using 10-fold cross-validation. The best performing models for ‘Mobility’ and ‘Self-care/Domestic Life’ achieved an F1-score of 0.80 using SVM with static word embeddings that were pre-trained on EHR for classifications within the ‘Mobility’ ICF chapter and pre-trained on SSA documentations for classifications within the ‘Self-care/Domestic Life’ ICF chapter.

## Methods

### Study Design

This study applied secondary analyses of a subset of data that was collected from caregivers of children and youth with and without craniofacial microsomia (CFM) as part of a longitudinal cohort study examining their prenatal risk factors and neurodevelopmental outcomes [35–41]. Ethical approval was originally obtained by the institutional review boards of Boston University and Seattle Children’s Hospital and then additional approval was obtained from the University of Illinois Chicago for secondary data analyses (protocol #2018 − 1273).

### Participants

The present analysis used data collected from caregivers of children and youth (11–17 years) with CFM or other childhood-onset disabilities, who were part of the second follow-up phase of a longitudinal cohort study. The parent study took place in 1996–2002 and enrolled caregivers of children with CFM who met the following inclusion criteria: (1) were younger than 36 months at the time of recruitment; (2) were not adopted; and (3) were diagnosed with CFM by a physician per established criteria for hemifacial microsomia, facial asymmetry, unilateral microtia, oculo-auriculo-vertebral syndrome, or Goldenhar syndrome. They were excluded if their child was diagnosed with chromosomal anomalies, Mendelian-inherited disorders, or in utero isotretinoin exposure [35, 36]. Caregivers of children without CFM were included if their child met the following inclusion criteria: (1) had no known birth defect; (2) was not adopted; and (3) was within two months of the age of children with CFM at the time of recruitment [35, 36]. A first phase of follow-up was conducted when the children were between ages 5 and 12 years. For the present study, participants were part of the second follow-up phase, when the children were aged between 11 and 17 years. Details about participant recruitment and enrollment in the parent and two follow-up phases are reported elsewhere [39, 41–43].

For this study, only children and youth with CFM or children and youth with other childhood-onset disabilities who receive services (i.e., physical therapy, speech language therapy, occupational therapy, visual therapy, hearing services, mental health services, other services, special education) were included because of previous evidence on their unmet participation need [42, 43]. This resulted in a total sample of 302 children and youth; 142 children and youth with CFM and 160 children and youth with other childhood-onset disabilities who receive services [39–41]. Participants with missing data on all strategies (*n* = 66) were excluded, resulting in a final sample of 236 participants.

### Measures

Caregivers completed the PEM-CY [44, 45] and as part of this administered up to 9 open-ended items about strategies they have used to support their child’s participation across activities in a specific setting (i.e., home, school, community) [44–47].

### Data Collection

**Fig. 1:**
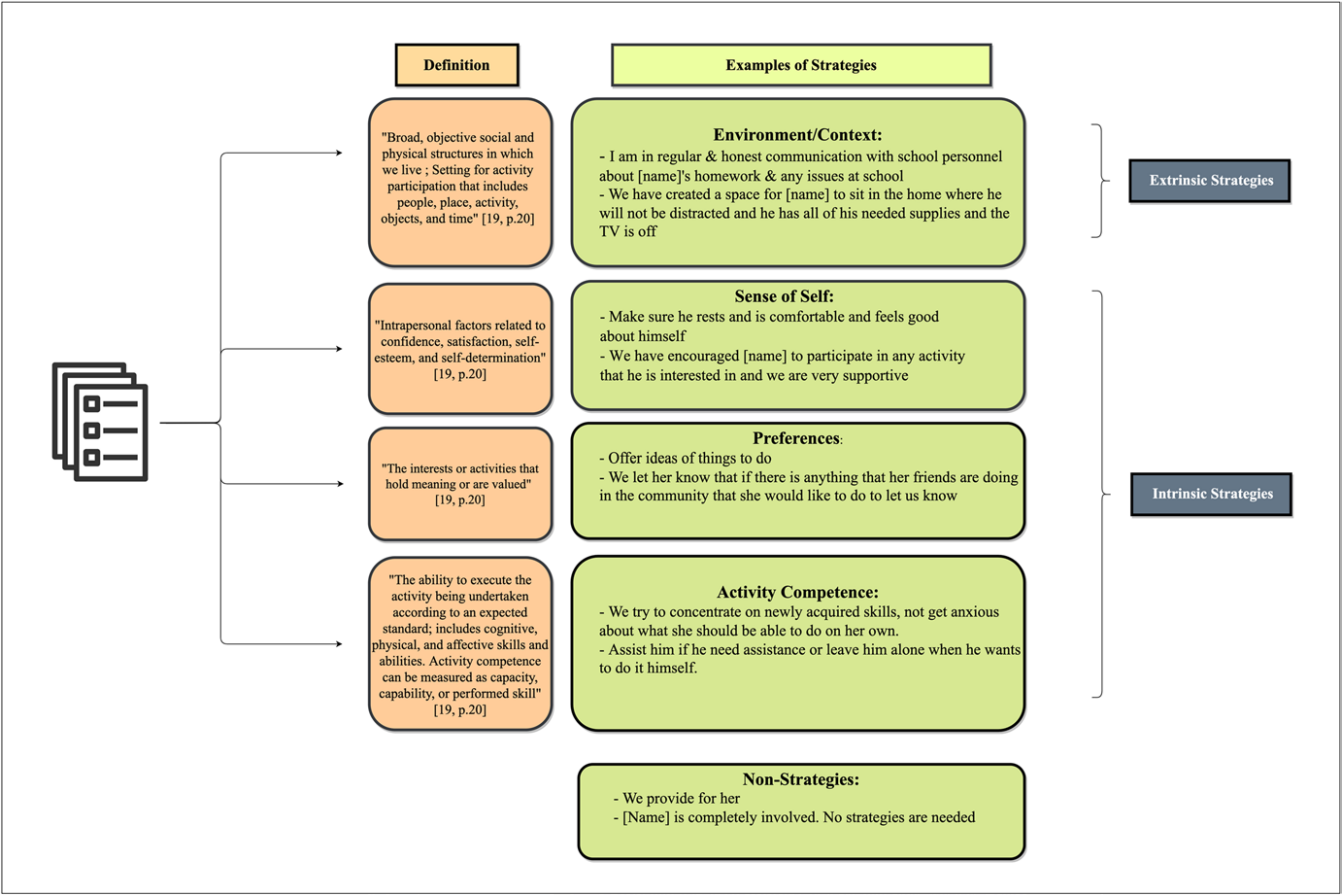
Definitions of classes with exemplar strategies

This study employs existing data on 1,576 participation-focused strategies from caregivers of children and youth (11–17 years) with CFM or other childhood-onset disabilities. Caregiver strategies for children and youth were annotated according to four classes representing participation-related constructs as outlined in the family of Participation-Related Concepts (fPRC) [20] that can be grouped in one extrinsic factor (i.e., environment/context) and three intrinsic factors (i.e., child or youth’s sense of self, preferences, activity competence), while filtering out non-strategies (e.g., “none”). Annotation was conducted by two native English speaking research assistants on a pre-occupational therapy track. Both assistants share expertise in the fPRC framework, and one assistant had prior experience in manual annotation of strategies data to guide development of the current PEM + strategy exchange feature [21]. Discrepancies between the two annotators were resolved through ‘majority rule’ involving a key informant who is an occupational therapist with expertise in child and youth participation as conceptualized by the fPRC. Details on the annotation process can be found elsewhere [21]. Figure 1 provides the definitions of the fPRC constructs and examples for each class. Number of records per class and percent agreement between annotators are summarized in Table 1. Annotator disagreements in classes with lower percent agreement (i.e., activity competence, preferences, non-strategy) were mainly with the environment/context class (i.e., 70% (16/23) for activity competence, 70% (14/20) for preferences, and 62% (18/29) for non-strategy).

**Table 1:** Number of records and percent agreement per class

Binary Classifications		
	#Records	Percent Agreement (%)
Strategy (vs. Non-Strategy)	1,470	96.07
Intrinsic (vs. Extrinsic)*	495	84.97
Multinomial Classification for Intrinsic Strategies*		
	#Records	Percent Agreement (%)
Sense of Self	307	82.08
Preferences	84	60.71
Activity Competence	104	68.27
Multinomial Classification into 5 classes		
	#Records	Percent Agreement (%)
Environment/Context	975	86.26
Sense of Self	307	82.08
Preferences	84	60.71
Activity Competence	104	68.27
Non-Strategy	106	58.49

Note. *Intrinsic strategies = strategies targeting a child or youth’s sense of self, preferences, or activity competence; extrinsic strategies = strategies targeting a child or youth’s environment/context

### Data Analyses

A series of analytic tasks were conducted using Python 3.8 [48] with the following libraries: Natural Language Toolkit (NLTK) [49] and Scikit-learn^1^.

#### Corpora Statistics

To describe the corpus (i.e., data in the form of a collection of texts), we calculated word statistics for the corpus, based on the vocabulary size (i.e., number of unique words in the corpus), total number of tokens (i.e., words), and the average strategy length (overall and per class).

#### Data Preprocessing

To prepare the data, we applied preprocessing methods including (1) case normalization; (2) spelling correction with an edit distance approach (i.e., minimum number of steps needed to transform one word into another) using a third-party library;^2^ (3) replacement of names and numbers using a named entity recognizer followed by manual checking (e.g., replace “Anna” with “[name]”); (4) removal of punctuation; and (5) removal of stop words such as “this” and “it” using a built-in stop words list in NLTK. The included text was normalized at the word level by applying lemmatization, in which words with the same root (e.g., sing and sung) are determined and mapped to their common lemma (e.g., sing).

#### Features

To classify the preprocessed caregiver strategies data (i.e., documents), features were designed by hand through careful examination of the training set and evaluated for their impact on class prediction across tested classifiers (i.e., naïve Bayes, logistic regression, SVM). Features included: (1) speech tags using Penn Treebank and dependency relation using universal dependency tags to identify syntactic patterns; (2) predefined likely sets of words for each of the four classes, by identifying words (w) in the training set most closely associated with each class (c) using Pointwise Mutual Information (PMI) [22]; and (3) dense lexicon features, as defined using concepts within the UMLS [50], a set of files and software combining health and biomedical vocabularies and standards. To manually map a related UMLS concept to strategies, we followed an iterative process of searching the UMLS, reading and re-reading the strategies, grouping strategies, drafting a title and description for the created groups, and assigning UMLS concepts to groups of strategies. The mapping was conducted by the first author (VK) with expertise in participation-focused pediatric rehabilitation. Mapping was discussed and refined during 5 meetings with a key informant (AB) with expertise in using the UMLS for classification tasks and during two additional meetings, which also involved another key informant (MK) with expertise in participation-focused pediatric rehabilitation.

$$\text{P}\text{M}\text{I} \left(\text{w},\text{c}\right)=log\frac{p\left(w,c\right)}{p\left(w\right)*p\left(c\right) }$$

#### Models

We experimented with three classification methods that are common in NLP tasks using smaller (n < 10k) datasets: (1) Naïve Bayes, (2) logistic regression, and (3) SVM. These approaches were chosen because of their different advantages. Logistic regression is more robust in its performance with correlated features, naïve Bayes can perform better with small datasets, and SVM is more resistant to overfitting, which is a risk when using small datasets [22, 51]. In addition, we included a baseline model, which served as a comparison for our results from classical models. For this, we applied a DummyClassifier^3^ using the “most_frequent” prediction method. This method always returns the most frequent class label and can be used to compare against other more complex classifiers (i.e., naïve Bayes, logistic regression, SVM).

We used a naïve Bayes algorithm, applying Laplace smoothing which is commonly used for naïve Bayes text classifications [22]. Naïve Bayes is a generative, probabilistic classifier based on two assumptions: (1) the position of the words does not matter, (2) the probabilities *P(f*_*i*_*|c)*, where *f*_*i*_ is a specific feature (e.g., a word in a bag-of-words model, or a manually engineered feature), are independent given the class c. Thus, features only encode word identity and not position [22]. Unknown words in the test set were removed.

We used logistic regression to train discriminative, probabilistic classifiers that make a decision about the class of a new input observation. This was done through learning from a training set a vector of weights and a bias term. The weight for a certain input represents how important that input feature is for the classification decision. A sigmoid function for binary problems and a softmax function for multinomial problems was used to transfer the calculated number expressing the weighted sum of the evidence for a class into the range (0, 1), which is needed for a probability and assignment of an input to a class. To train the system and find the optimal weights that maximize the probability of the correct class, the cross-entropy loss and stochastic gradient descent computed over batches of training instances (max_iter = 100) was used. L2 regularization was applied to prevent overfitting of the model (i.e., a model that fits the training data too well and, therefore, learns to place high weights on irrelevant characteristics that do not generalize to the test set or other datasets).

We used a SVM algorithm with a linear Kernel [51], which finds a hyperplane that best divides data into classes. Compared to logistic regression that defines a separating line based on all available data points, SVM defines a separating hyperplane based on data points that are identified as more important than others (i.e., support vectors that are located closer to the hyperplane). The hyperplane serves as a decision boundary (maximum margin separator) and is chosen to be farthest away from the support vectors by minimizing the expected generalization loss on the training data [51].

We used two common ways to represent the documents (i.e., caregiver strategies) as numeric vectors to perform calculations: (1) We encoded the documents using a term frequency-inverse document frequency (TF-IDF) [22] feature representations with a vocabulary size of the 5000 most frequent words within our dataset; and (2) converted documents into sentence embeddings using Doc2Vec [52] with a vector size of 100, a minimum count of 1, and epochs (i.e., complete passes of the training data through the algorithm) of 30. Through TF-IDF, the relative importance of words in documents (i.e., caregiver strategies) is reflected whereas with Doc2Vec, sentence embeddings also include documents’ semantic information and enable capturing of different relations between words such as synonyms or antonyms. The same two approaches were used to convert UMLS concepts (e.g., “Adjustment of physical environment”) into vectors to then include into the analyses. Two feature vectors were constructed by concatenating the manually created features (i.e., speech tags, universal dependency tags, predefined likely sets of words) with: (1) the TF-IDF encoded caregiver strategies and UMLS concepts, and (2) the sentence embeddings using Doc2Vec for the caregiver strategies and UMLS concepts.

To analyze the data, the dataset was divided into a fixed training set (80% of the data) and a test set (20% of the data). Within the fixed training set, repeated stratified 10-fold cross-validation^4^ was applied to train the classifiers and optimize model parameters, as suggested for small sample sizes [22, 53]. The resulting best model was then evaluated on the test set to report on model performance. A final test set to report on model performance ensures robustness of the approach [22].

#### Experimental Setting

**Fig. 2:**
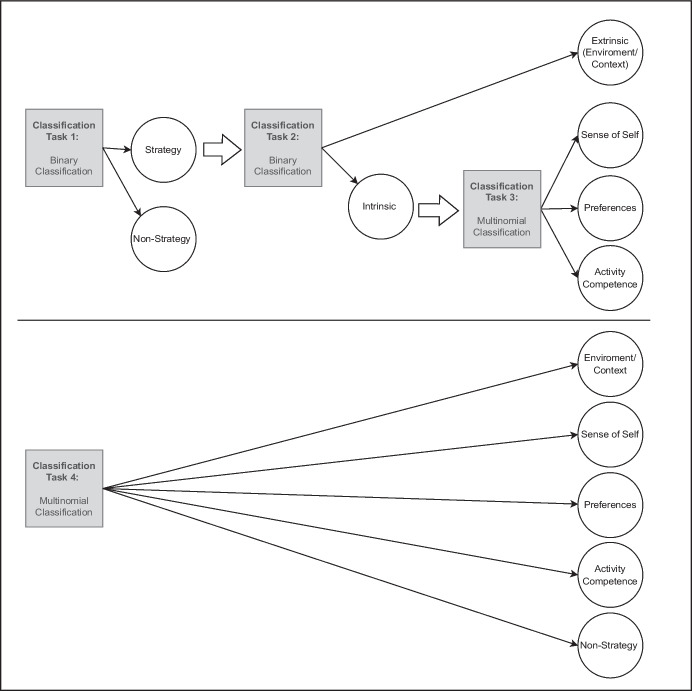
Four classification tasks

A series of binary and multinomial supervised machine learning models with increasing complexity was applied with the resulting sentence embeddings from caregiver strategies and with the constructed feature vectors (see Fig. 2): For classification task 1, we applied binary classifiers to group records into documents that classify as strategies (e.g., “I have [name] put music on to help her focus”) and documents that do not classify as strategies (e.g., “none”). For classification task 2, we applied binary classifiers to further group documents that classify as caregiver strategies into extrinsic (i.e., participation-related construct ‘environment/context’) and intrinsic (i.e., participation-related constructs ‘sense of self’, ‘preferences’, ‘activity competences’) strategies. For classification task 3, we applied multinomial classifiers to further group intrinsic strategies into their containing fPRC participation-related constructs (i.e., sense of self, preferences, activity competences). For classification task 4, we applied multinomial classifiers to group records into five classes (i.e., environment/context, sense of self, preferences, activity competences, non-strategy).

#### Evaluation

To evaluate the text classification, we calculated accuracy, precision, recall, and F1 for each class separately and across classes using macro-averaging [22]. Accuracy is a metric for correct predictions (i.e., correct predictions out of total), precision measures model exactness (i.e., portion of correct positive predictions), recall measures model sensitivity (i.e., portion of correctly predicted positives out of all actual positives), and F1 is a metric that incorporates precision and recall. Subsequent analyses were performed to examine the relevance of included features for predicting the classes.

$$\text{F}1 \ \text{score}=2\ast\frac{Precision\ast Recall}{Precision+Recall}$$

## Results

### Sample Characteristics

The sample included 236 children and youth with CFM or with other childhood-onset disabilities, between 11 and 17 years old (*M* = 13.42) (see Table 2). More than half of the included children and youth were male and White, and of parents with a high school/general education diploma (GED) or some college/associated degree. Speech therapy was the most common received type of service among included children and youth.

**Table 2:** Family and child/youth characteristics and service use

Characteristics and Service Use	*n* = 236
Child/youth gender (Male), *n* (%)^†^	133 (56.36)
Children and youth with CFM	109 (46.19)
Children and youth with other childhood-onset disabilities	127 (53.81)
Child/youth age in years, *M (SD)*	13.42 (1.40)
Child/youth race and ethnicity, *n* (%)^†^	
White, Non-Latinx	195 (82.63)
White, Latinx	22 (9.32)
Black	7 (2.97)
Other	12 (5.08)
Caregiver education^†^	
High school/GED or some college/associated degree	103 (44.98)
Bachelor’s/college degree	79 (34.50)
Graduate degree	47 (20.52)
Type of service received, *n* (%)^†§^	
Occupational therapy	43 (18.45)
Physical therapy	77 (32.91)
Speech therapy	107 (46.12)
Vision therapy	29 (12.34)
Hearing services	39 (16.67)
Mental health services	52 (22.13)
Special education	76 (32.76)
Other services	83 (35.47)

Note. CFM = Craniofacial microsomia; GED = General education diploma

^†^ = missing data

§ = multiple responses possible

### Corpora Statistics

The used corpus consists of 1,576 documents (i.e., caregiver strategies) and a total of 10,804 words with a vocabulary size of 2,337. The length of a caregiver strategy ranged from 1 to 32 words (*M =* 6.86). The average length of caregiver strategies was similar and greater than 5 words across classes (i.e., 7.20 words for strategies targeting the environment/context; 6.60 words for strategies targeting child or youth sense of self; 7.64 words for strategies targeting child or youth preferences; 5.85 words for strategies targeting child or youth activity competence; 5.21 words for strategies that did not classify as strategies (i.e., no adaptation described)).

### UMLS Representation

The 1,576 caregiver strategies were clustered to 71 concepts (i.e., 38 for environment/context, 15 for sense of self, 12 for preferences, 6 for activity competence). A total of 49 of the 71 concepts could be mapped to existing UMLS concepts stemming from 36 different ontologies (see Appendix 1). The most prevalent ontologies were Systematized Nomenclature of Medicine United States (SNOMED_US) (*n* = 15), Consumer Health Vocabulary (CHV) (*n* = 15), Nursing Outcome Classification (NOC) (*n* = 11), Psychological Index Terms (PSY) (*n* = 11), Nursing Intervention Classification (NIC) (*n* = 10), and Read Codes (RCD) (*n* = 10). For 13 of the 49 UMLS concepts, a definition was available within UMLS and only 5 UMLS concepts indicated family member involvement (e.g., “parenting – offers child choices”). A total of 22 concepts (i.e., 12 for environment/context, 6 for sense of self, 3 for preferences, 1 for activity competence; see Appendix 1) could not be mapped to any existing UMLS concept.

### Model Performance

#### Classification Task 1. Binary Classification of Records to Filter Out Non-Strategies

We first applied binary classification to test the automated grouping of records into “caregiver strategies” and “no caregiver strategies”. Of the three tested classifiers, SVM performed best when including manually created features and TF-IDF encoded strategies and UMLS concepts (accuracy = 94.92; F1 = 0.70; see Table 3). This was 1.59% higher accuracy than the baseline algorithm (accuracy = 93.33%). Misprediction for the SVM occurred mainly for the non-strategy class (*n* = 15/21; see Appendix 2). Subsequent analyses on relevance of included features for predicting the classes revealed no single best performing feature (see Table 4).

#### Classification Task 2. Binary Classification of Caregiver Strategies into Extrinsic and Intrinsic Strategies

Records that are considered as caregiver strategies (i.e., excluding records that do not classify as a caregiver strategy) underwent further testing by applying binary classifiers to map caregiver strategies to extrinsic (i.e., strategies targeting the environment/context) and intrinsic strategies (i.e., strategies targeting a child’s sense of self, preferences, activity competences). SVM was the best performing model (accuracy = 85.71%; F1 = 0.83), when only including the TF-IDF encoded caregiver strategies without added features. It had 19.38% higher accuracy when compared to the baseline model (accuracy = 66.33%). Mispredictions for the SVM model occurred mainly for intrinsic strategies (i.e., strategies targeting a child’s sense of self, preferences, activity competence) (*n* = 30/99; see Appendix 2).

#### Classification Task 3. Multinomial Classification of the Intrinsic Strategies into the 3 fPRC Participation-Related Constructs

We applied multinomial classifiers to test the further classification of intrinsic caregiver strategies into 3 fPRC-related constructs (i.e., sense of self, preferences, activity competence). SVM was the best performing model when only including the TF-IDF encoded caregiver strategies without added features (accuracy = 83.84; F1 = 0.80; see Table 3). This was an increase of 22.22% in accuracy when compared to the baseline model (accuracy = 61.62%). Mispredictions for the SVM model occurred mainly for the class representing caregiver strategies targeting a child’s preferences (*n* = 6/17; see Appendix 2).

#### Classification Task 4. Multinomial Classification into the 4 fPRC Participation-Related Constructs and a Non-Strategy Class

We applied multinomial classifiers to test the classification of the records into all five classes (i.e. environment/context, sense of self, preferences, activity competence, non-strategy). SVM was the best performing model when only including the TF-IDF encoded caregiver-strategies without added features (accuracy = 78.10; F1 = 0.58; see Table 3). This was an accuracy increase of 16.19% when compared to the baseline model (accuracy = 61.91). Mispredictions for the SVM model occurred mainly for the non-strategy class (*n* = 18/21; see Appendix 2).

**Table 3:** Model performance by classification task

1. Binary classification of records to filter out non-strategies								
	Embeddings, TF-IDF		Added Features, TF-IDF		Embeddings, Doc2Vec		Added Features, Doc2Vec	
	Accuracy (%)	F1	Accuracy (%)	F1	Accuracy (%)	F1	Accuracy (%)	F1
LR	93.33	0.48	94.60	0.67	93.33	0.48	94.29	0.67
NB	93.33	0.48	93.65	0.53	93.33	0.48	94.29	0.67
SVM	93.33	0.53	**94.92**	**0.70**	93.33	0.48	93.97	0.67
2. Binary classification of caregiver strategies into extrinsic and intrinsic strategies								
	Embeddings, TF-IDF		Added Features, TF-IDF		Embeddings, Doc2Vec		Added Features, Doc2Vec	
	Accuracy (%)	F1	Accuracy (%)	F1	Accuracy (%)	F1	Accuracy (%)	F1
LR	84.01	0.80	74.49	0.69	68.71	0.49	80.95	0.77
NB	82.31	0.77	73.47	0.62	70.41	0.64	75.51	0.72
SVM	**85.71**	**0.83**	75.85	0.69	66.33	0.40	84.69	0.82
3. Multinomial classification of the intrinsic strategies into the 3 fPRC participation-related constructs								
	Embeddings, TF-IDF		Added Features, TF-IDF		Embeddings, Doc2Vec		Added Features, Doc2Vec	
	Accuracy (%)	F1	Accuracy (%)	F1	Accuracy (%)	F1	Accuracy (%)	F1
LR	79.80	0.70	74.75	0.67	61.62	0.25	76.77	0.29
NB	69.70	0.45	68.96	0.43	61.62	0.25	41.41	0.20
SVM	**83.84**	**0.80**	74.75	0.65	61.62	0.25	73.74	0.28
4. Multinomial classification into the 4 fPRC participation-related constructs and a non-strategy class								
	Embeddings, TF-IDF		Added Features, TF-IDF		Embeddings, Doc2Vec		Added Features, Doc2Vec	
	Accuracy (%)	F1	Accuracy (%)	F1	Accuracy (%)	F1	Accuracy (%)	F1
LR	77.14	0.48	68.25	0.48	61.91	0.15	75.56	0.57
NB	69.52	0.27	65.40	0.24	61.91	0.15	62.22	0.49
SVM	**78.10**	**0.58**	68.89	0.49	61.91	0.15	77.14	0.57

Note. LR = logistic regression; NB = naïve Bayes; SVM = support vector machines; fPRC = family Participation-Related Constructs

Estimates for best performing model per classification task are in bold

**Table 4:** Feature performance of best performing model for classification task 1

	Classification Task 1: Binary (Strategy/Non-Strategy)	
	Accuracy (%)	F1
Best performing model	**94.92**	**0.70**
Added UMLS	93.97	0.57
Added speech tagging	93.97	0.57
Added dependency parsing	93.97	0.57
Added likely sets of words	93.33	0.56

UMLS = Uniform Medical Language System

## Discussion

The use of AI might be one way to further customize participation-focused pediatric rehabilitation interventions such as PEM + to individual needs of children, youth and their families [13–15]. To our knowledge, this is one of the first studies to apply NLP and use of the UMLS for customizing participation-focused pediatric rehabilitation services [15, 16]. We conducted a series of classification tasks with increased complexity to identify best performing predictive models to classify caregiver strategies to key predictors of participation. We manually created features, including UMLS concepts, to support these classification tasks. Results can inform the integration of such predictive models into a strategy exchange feature within PEM + to support families in finding and selecting suitable strategies for goal attainment.

SVM was the best performing model for all four classification tasks, achieving an accuracy of 78.10 − 94.92%, a F1 score of 0.58–0.83 and an accuracy increase of 1.59 − 22.22% when compared to the baseline models using a dummy classifier. Our F1 scores for classifying caregiver strategies into more fine-grained constructs (i.e., classification task 2: intrinsic/extrinsic strategies; classification task 3: 4 fPRC participation-related constructs) were similar to prior research classifying narrative data to ICF codes such as the fPRC participation-related construct ‘activity competence’ (e.g., hand and arm use, changing basic body position) (F1 = 0.80–0.85) [26–28]. However, the two classification tasks that included a non-strategy class (i.e., classification task 1: strategy/non-strategy; classification task 4: 5-class classification) had lower model performance, with most mispredictions occurring in the non-strategy class, which arguably contains the highest diversity of strategies (e.g., “none” or “[name] is completely involved. No strategies are needed”). In addition, both models were created with an imbalanced dataset (i.e., classification task 1: 1,470 strategies; 106 non-strategies; classification task 4: 495 strategies for environment/context; 307 strategies for sense of self; 84 strategies for preferences; 103 strategies for activity competence; 106 non-strategies) with relatively few instances in the non-strategy class, potentially contributing to lower increase in model performance when compared to the baseline model.

Interestingly, our manually created features only improved model performance for classification task 1, indicating that the added features may be more useful for separating records into strategies and non-strategies versus more fine-grained classifications of types of strategies. This result might be explained by more pronounced semantic differences (e.g., frequency of one-word responses) among strategies and non-strategies as compared to types of strategies. The lack of increased model performance through added features, in particular the UMLS feature, is surprising and may reflect the relatively high number of strategies without fitting UMLS concepts, which in turn might be a result of the medical focus (e.g., body structures and functions) of most terminologies within the UMLS. However, the ICF [54] and its children and youth version (ICF-CY) [55] that represent a biopsychosocial mindset and include constructs more proximal to participation were only represented once among the mapped UMLS concepts. This might be due to prior stated limitations when using the ICF and ICF-CY, such as a lack of comprehensiveness [56] and overlaps among ICF codes [27, 57] particularly for participation-related concepts (i.e., activity competence) and participation [20, 57]. Taken together, the current UMLS might be more suited to support the classification of medical concepts such as body structures and functions as compared to participation and its related constructs. Future work should focus on expanding existing terminologies to include concepts that are more proximal to participation [27, 28, 58].

Overall, results provide evidence for integrating NLP application to customize the functionality of participation-focused pediatric rehabilitation interventions like PEM + among caregivers who complete the PEM-CY. More specifically, results suggest overall higher accuracy for the pipelined classification approach (i.e., classification tasks 1–3, reaching accuracy between 83.84 and 94.92% (mean accuracy = 88.16%) and F1 scores of 0.70–0.83) as compared to the 5-class classification (i.e., classification task 4, reaching 78.10% accuracy and an F1 score of 0.58), therefore, supporting its implementation into PEM+. The lower model performance for the 5-class classification may also reflect our drop in annotator agreement with increased complexity. Similar to annotator disagreements, which were mostly related to environment/context, mispredictions were also mostly related to the environment/context class (see Appendix 2), confirming the complexity of participation and its related constructs [20]. Nevertheless, future research should focus on designing an algorithm that successfully classifies records into all five classes, to further facilitate its implementation into the end-to-end PEM system. One way might be by applying BERT [31] which incorporates contextualized word embeddings such as done in other clinical text classification [27, 28]. Such research is underway to explore and expand beyond the application of classical methods. In addition, future research could focus on exploring the adaptation and extension of PEM + for use with self-report versions of the PEM that are under development, given the promising performance of classifying PEM-CY-generated caregiver strategies to key predictors of participation.

Results of this research should be interpreted in light of some limitations. We used an unbalanced and small dataset consisting of 1,576 strategies, however, representing a diverse sample of caregivers with respect to their educational background and, therefore, providing evidence for applicability of the strategy classifications to improve the PEM + strategy exchange feature for caregivers with differing levels of health literacy. Future work should focus on diversifying the sample also in terms of race and ethnicity while considering multiple geographical regions and clinics and creating a larger and more balanced dataset to improve overall model performance.

## Conclusion

This research extends existing evidence on the use of NLP in participation-focused pediatric rehabilitation interventions. We have presented a pipeline of three classification tasks as well as a multinomial classification with 5-classes to group caregiver strategies into four participation-related constructs and a non-strategy class. Model performance for the three pipelined classification tasks reached encouraging accuracy and macro-averaged F1-scores, laying groundwork for the use of NLP when classifying caregiver strategies to support child and youth participation in daily activities. Future research should focus on expanding existing terminologies to include concepts that are more proximal to participation and are under-studied within NLP [26–28, 58]. Additionally, future research would benefit from a larger and more balanced dataset (i.e., even numbers of documents per class) of participation-focused caregiver strategies.

### Supplementary Information

Below is the link to the electronic supplementary material.

ESM 1 (DOCX 34.1 KB)

ESM 2 (DOCX 17.0 KB)

## Data Availability

Data available on request from the authors.

## Footnotes

1 https://scikit-learn.org/stable/.

2 https://github.com/barrust/pyspellchecker.

3 https://scikit-learn.org/stable/modules/generated/sklearn.dummy.DummyClassifier.html.

4 https://scikit-learn.org/stable/modules/generated/sklearn.model_selection.RepeatedStratifiedKFold.html.
