# Supplementary material for: Natural Language Processing to Classify Caregiver Strategies Supporting Participation Among Children and Youth with Craniofacial Microsomia and Other Childhood-Onset Disabilities
Source: J Healthc Inform Res. 2023 Sep 18;7(4):480–500. doi: 10.1007/s41666-023-00149-y (PMC10620347; doi:10.1007/s41666-023-00149-y)
Supplement: Supplementary file 1 — (DOCX 34.1 KB) [file 41666_2023_149_MOESM1_ESM.docx]

Appendix 1: UMLS Concepts and Terminologies

|  | **Concept** | **Created Description for Concept** | **UMLS Concept with CUI** | **Terminology/**  **Ontology** |
| --- | --- | --- | --- | --- |
| Environment/Context | | | | |
| 1 | Parents provide ride | Definition: Parents drive their child somewhere [Transportation happens by car] | UMLS: Non-emergency transportation, per mile - vehicle provided by individual (family member, self, neighbor) with vested interest (C0497714) | HCPCS |
| 2 | Parents bring child somewhere | Definition: Parents bring their child to an activity [not clear how; whether by car or otherwise] | UMLS: None |  |
| 3 | Parents provide transportation | Definition: Parents provide transportation to their child [not clear whether by car or otherwise] | UMLS: Transportation (C0040756) | MTH, MSH, NCI, NCI_caDSR, CHV, HL7V3.0, OMS, PSY |
| 4 | Parents support with money | Definition: Parents support their child’s participation financially (e.g., by buying materials, paying fees) | UMLS: Financial Support (C0016118) | MSH, CHV, MSHPOR, MSHSPA, MSHCZE, MSHDUT, MSHSWE, MSHNOR |
| 5 | Parents support with providing supplies | Definition: Parents provide supplies (e.g., equipment, school supplies, arts and craft material) to support their child’s participation. | UMLS: Providing material (C0557038) | SNOMEDCT_US, RCD, SCTSPA |
| 6 | Parents volunteer | Defintion: Parents volunteer at events or for other activities (or volunteer together with child) to support their child’s participation within the same activity.  Note: Does not contain strategies about volunteering at school | UMLS: Participates as a volunteer (C1821911) | NOC |
| 7 | Parents volunteer at school | Definition: Parents volunteer at school to support their child’s participation at school. Note: School/activity specific to school must be mentioned | UMLS: school volunteer (C0682222) | AOD |
| 8 | Parents participate in activities together with child | Definition: Parents participate together with their child in non-specified or school activities to support their child’s participation (e.g., they attend activities or intentionally do things with the whole family) | UMLS: Participates in activities together (C1820598) | NOC |
| 9 | Family eats together | Defintion: Family eats together to support child’s participation | UMLS: Eats meals together (C4716091) | NOC |
| 10 | Parents participate in leisure-activities together with child | Definition: Parents participate together with their child in leisure activities (e.g., they attend activities or intentionally do things with the whole family) Note: Leisure activity needs to be stated in the strategy. | UMLS: Participates in leisure-time activities together (C4715990) | NOC |
| 11 | Parents provide opportunities | Definition: Parents provide, explore or look for opportunities for their child to participate. | UMLS: Provide opportunities for child to have normal childhood experiences (C0514950) | NIC |
| 12 | Parent provides access to an activity | Defintion: Parents take their child to an activity or enroll their child in an activity or provide access to this activity. | UMLS: None |  |
| 13 | Parents provide activities that encourage interaction with other children | Definition: Parents organize or enable social activities to support their child’s participation. | UMLS: Provide activities that encourage interaction among children (C0514618) | NIC |
| 14 | Parents stay updated/informed | Defintion: Parents stay updated to support their child’s participation. | UMLS: Parent informed (C0809938) | CCPSS |
| 15 | Parents are in contact with teachers/leaders | Definition: Parents communicate with teachers/leaders of community programs and/or build relationship with them. | UMLS: None |  |
| 16 | Parents inform child about what will happen next | Definition: Parents support their child’s participation by informing their child about what will happen next. | UMLS: None |  |
| 17 | Parents support through providing structure | Definition: Parents support their child’s participation by organizing, structuring daily life and by planning ahead (e.g., providing schedules, lists, plans, adjust timing) | UMLS: Maintain structured environment and routines (i.e., ensure consistent daily schedules; provide frequent reminders; and provide calendars and other environmental cues) (C4710380) | NIC |
| 18 | Parents support with reminders | Defintion: Parents support child’s participation with reminders | UMLS: Reminder (C1709896) | NCI |
| 19 | Parents support child’s participation by establishing routines | Definition: Parents support child’s participation by establishing/following routines (e.g., do things daily) | UMLS: Routine (C0205547) | MTH, SNOMEDCT_US, NCI, CHV, LNC, SNMI, RCD, SCTSPA |
| 20 | Parents involve babysitter | Definition: Parents involve a babysitter to support their child’s participation | UMLS: Babysitter (C0337606) | SNOMEDCT_US, CHV, PSY, SNMI, SCTSPA |
| 21 | Parents involve a tutor | Definition: Parents involve a tutor or aid teacher to support their child’s participation | UMLS: Tutors (C0871554) | MTH, CHV, PSY |
| 22 | Parents involve another family member | Definition: Parents have support from a family member who supports their child’s participation | UMLS: Help by relatives (C0581904) | SNOMEDCT_US, RCD, SCTSPA |
| 23 | Parents involve another parent friend or other adult person | Defintion: Parents involve another parent or other adult to support their child’s participation | UMLS: Support from friends (C4708444) | MTH, NOC |
| 24 | Parents involve sibling | Definition: Parents involve a sibling to support their child’s participation | UMLS: None |  |
| 25 | Parents involve another child/friend | Definition: Parents find or involve another child or friend to support their child’s participation | UMLS: Provide opportunities for peer group support (C0514955) | NIC |
| 26 | Parents build relationships with neighbors |  | UMLS: Relationship with neighbors (C0557151) | SNOMEDCT_US, RCD, RCDAE, SCTSPA |
| 27 | Parents build relationships with friends/other parents |  | UMLS: Relationships with friends (C0516939) | NOC |
| 28 | Parents use distraction or redirect attention | Definition: Parents use toys or other ways to distract their child or redirect their child’s attention to support their child’s participation | UMLS: Shifting attention (C2370875) | ICF, ICF-CY |
| 29 | Parents bring or use items that help | Definition: Parents bring or use items (e.g., toy, snack) that support their child’s participation | UMLS: None |  |
| 30 | Parents provide space, change location or arrange items | Definition: Parents provide space, change location (e.g., room or residence) or arrange items to support child participation | UMLS: None |  |
| 31 | Parents provide optimal environment | Definition: Parents provide an optimal environment (e.g., good lighted/quiet environment/time) to support their child’s participation | UMLS: Environment: Physical surroundings (e.g., lighting, noise) (C4035960) | LNC |
| 32 | Parents provide adjusted physical environment/equipment | Definition: Parents provide child-sized, child-friendly equipment to support their child participation | Adjustment of physical environment (C4304983) | SNOMEDCT_US, SCTSPA |
| 33 | Parents support through music | Defintion: Parents support through the use of music or through singing | UMLS: Benefits of music (C4717011) | NOC |
| 34 | Parents provide time/full attention | Definition: Parents are available, have time and provide their child their full attention, invest time in the child | UMLS: None |  |
| 35 | Parents make sure child has had enough sleep and food | Definition: Parents make sure their child is well rested and fed to support their participation. | UMLS: None |  |
| 36 | Parents limit or restrict TV/media | Definition: Parents provide media/TV fee time to support their child’s participation | UMLS: None |  |
| 37 | Parents model the activity | Definition: Parents model the activity/show how do do the activity so that the child can imitate. | UMLS: None |  |
| 38 | Parents give cues | Definition: Parents give their children cues including visuals to support their participation. | UMLS: None |  |
| Sense of Self | | | | |
| 39 | Parents encourages child | Definition: Parents encourage/motivate child an provide positive reinforcement (e.g., praise) to support their participation | UMLS: Provide positive reinforcement for participation in activities (C0515032) | NIC |
| 40 | Parents builds on strengths and supports effort | Definition: Parents build on their child’s strengths, support their child’s efforts, focus on the positive, and support their child’s participation with confidence boosters. | UMLS: Emphasize child's strengths (C0510546) | NIC |
| 41 | Parents ask about day and listen | Definition: Parents ask about the child’s day or activities and support open communication, frequent talking frequently and listen. | UMLS:  Facilitate open communication among family members (C0511328) | NIC |
| 42 | Support | Definition: Parents support child in their efforts, provide cognitive/emotional support and support their activities to support their participation | UMLS: Parental support (C0418946) | SNOMEDCT_US, RCD, AOD, SCTSPA |
| 43 | Parents include the child into the full activity | Definition: Parents include their child, they let their child be more involved, they invite their child to join, and have their child helping them | UMLS: None |  |
| 44 | Parents let child volunteer | Definition: Parents let their child volunteer to support their child’s participation | UMLS: None |  |
| 45 | Parent asks child to help the caregiver or others | Definition: Parents ask child for help or invite child to help | UMLS: None |  |
| 46 | Parents do not treat child differently | Definition: Parents do not treat child differently to support their child’s participation | UMLS: None |  |
| 47 | Parents provides comfort to the child | Definition: Parents provide comfort to their child to support their child’s participation | UMLS: Actual Positive Comfort (C2712134) | MTH, ICNP |
| 48 | Parents are interested in the child | Definition: Parents show interest in their child to support their participation | UMLS: None |  |
| 49 | Parents support their child’s self-motivation and problem-solving | Definition: Parents support their child’s self-motivation and problem-solving to support their child’s participation | UMLS: Problem solving in children (C3825791) | LCH_NW |
| 50 | Parents support by providing goals |  | UMLS: Sets goals (C0150598) | MTH, SNOMEDCT_US, NCI, NCI_CTRP, CHV, NOC, PSY |
| 51 | Parent provides love |  | UMLS: Love (C0024028) | MSH, SNOMEDCT_US, NCI, CHV, PSY, LCH_NW, LCH, SNMI |
| 52 | Parent makes sure child feels confident in taking on a task | Definition: Parents make sure child understands tasks and feels secure with instructions given, | UMLS: Self Confidence (C0237529) | MTH, MSH, SNOMEDCT_US, NCI, CHV, NOC, PSY, RCD |
| 53 | Parent advocates for child |  | UMLS: None |  |
| Preferences | | | | |
| 54 | Parents suggest ideas | Definition: Parents offer or suggest ideas to support their child’s participation | UMLS: None |  |
| 55 | Parents offer choices | Definition: Parent offers options to choose from to support their child’s participation | UMLS: parenting - offers child choices (C3862624) | MEDCIN |
| 56 | Parents inquire about child’s interests | Definition: Parents ask their child about their interests or inquire about their interests to support their child’s participation | UMLS: Level of interest (C0424090) | SNOMEDCT_US, CHV, PSY, RCD, SCTSPA |
| 57 | Parents make the activity fun | Definition: Parents make the activities fun and support their child in their clear interests by doing fun games and things that the child likes | UMLS: None |  |
| 58 | Parents laugh with child |  | UMLS: Laughter (C0023133) | MSH, SNOMEDCT_US, MDR, CHV, PSY, LCH_NW, LCH, SNMI |
| 59 | Parents use reward systems | Definition: Parents use reward systems or incentives to support their child’s participation | UMLS: Establish incentive and reward systems (C0677344) | PPAC |
| 60 | Parents use bargaining | Definition: Parents use bargaining to support their child’s participation | UMLS: bargaining (C0871661) | CHV, PSY, AOD |
| 61 | Parents set rules | Definition: Parents set rules or boundaries to support their child’s participation | UMLS: Establishes family rules (C1820601) | NOC |
| 62 | Parent sets clear expectations and consequences | Definition: Parents communicate or set clear expectations(e.g., by telling the child or by demonstrating) to support their child’s participation | UMLS: Communicate rules, behavioral expectations, and consequences using simple language with visual cues, as necessary (C0509555) | NIC |
| 63 | Parent forces child | Definition: Parents force their child to support their child’s participation | UMLS: Forced (C0443221) | MTH, SNOMEDCT_US, NCI, CHV, RCD, SCTSPA |
| 64 | Parent punishes child | Definition: Parent punishes their child to support their child’s participation | UMLS: Punishment (C0034119) | MSH, NCI, CHV, PSY, LCH_NW, LCH, CSP, MSHPOR |
| 65 | Parent nags | Definition: Parent nags to support their child’s participation | UMLS: None |  |
| Activity Competence | | | | |
| 66 | Parent is helping the child with an activity | Definition: Parents are helping/assisting their child with an activity to support their child’s participation | UMLS: Assisting with functional activity (C0582444) | SNOMEDCT_US, RCD, SCTSPA |
| 67 | Answer questions | Definition: Parents answer questions of the child | UMLS: Answer questions and discuss concerns, as appropriate (C0508433) | NIC |
| 68 | Parent is supporting by making child practice, repeat, imitate an activity |  | UMLS: None |  |
| 69 | Parent teaches child an activity | Definition: Parents support their child’s participation by guiding and coaching the child and by teaching the child how to perform an activity, review assignments with the child | UMLS: Teaching of skills (C0557047) | CHV, RCD, SNOMEDCT_US, SCTSPA |
| 70 | Parent concentrates on newly acquired skills | Definition: Parent concentrates on newly acquired skills, make sure child knows how to do an activity to support their participation. | UMLS: Skills training in activities related to learning (C0846678) | ICD10AM |
| 71 | Parent support child to do an activity independent |  | UMLS: Independence (C0085862) | MTH, NCI, CHV, LNC, NOC, PSY |

*Note.* HCPCS = Healthcare Common Procedure Coding System; MTH = Metathesaurus Names; MSH = MeSH; NCI = National Cancer Institute; NCI_caDSR = Cancer Data Standards Registry and Repository; CHV = Consumer Health Vocabulary; HL7V3.0 = HL7 Version 3.0; OMS = Omaha System; PSY = Psychological Index Terms; MSHPOR = MeSH Portuguese; MSHSPA = MeSH Spanish; MSHCZE = MeSH Czech; MSHDUT = MeSH Dutch; MSHSWE = MeSH Swedish; MSHNOR = MeSH Norwegian; SNOMEDCT_US = Systematized Nomenclature of Medicine United States; RCD = Read Codes; NOC = Nursing Outcome Classification; AOD = Alcohol and Other Drug Thesaurus; NIC = Nursing Intervention Classification; CCPSS = Clinical Problem Statements; LNC = LOINC; SNMI = Systematized Nomenclature of Medicine Intl 1998; SCTSPA = Systematized Nomenclature of Medicine - Clinical Terms Spanish Edition; ICNP = International Classification for Nursing Practice; RCDAE = Read Codes American English; ICF = International Classification for Functioning, Disability and Health; ICF-CY = International Classification for Functioning, Disability and Health for Children and Youth; LCH_NW = Library of Congress Subject Headings, Northwestern University subset; LCH = Library of Congress Subject Headings; NCI_CTRP = Clinical Trials Reporting Program Terms; MDR = MedDRA; PPAC = Pharmacy Practice Activity Classification; CSP = CRISP Thesaurus; ICD10AM = International Classification of Diseases and Related Health Problems, Tenth Revision, Australian Modification

**Article title**: Natural Language Processing to Classify Caregiver Strategies Supporting Participation Among Children and Youth with Craniofacial Microsomia and Childhood-Onset Disabilities

**Journal name**: Journal of Healthcare Informatics Research.

**Author names and affiliations:** Vera C Kaelin [1, 2], Andrew D Boyd [1, 3], Martha M Werler [4], *Natalie Parde [5, 6], *Mary A Khetani [1, 2, 7, 8]

[1] Rehabilitation Sciences, University of Illinois Chicago, Chicago, USA

[2] Children’s Participation in Environment Research Lab, University of Illinois Chicago, Chicago, USA

[3] Biomedical and Health Information Sciences, University of Illinois Chicago, Chicago, USA

[4] Epidemiology, Boston University, Boston, USA

[5] Computer Science, University of Illinois Chicago, Chicago, USA

[6] Natural Language Processing Laboratory, University of Illinois Chicago, Chicago, USA

[7] CanChild Centre for Childhood Disability Research, McMaster University, Hamilton, CA

[8] Occupational Therapy, University of Illinois Chicago, Chicago, USA

* Co-Senior and Co-Corresponding authors

**Corresponding authors**: Mary A. Khetani, email: [mkhetani@uic.edu](mailto:mkhetani@uic.edu); Natalie Parde, email: [parde@uic.edu](mailto:parde@uic.edu)
