# Supplementary material for: Natural Language Processing to Classify Caregiver Strategies Supporting Participation Among Children and Youth with Craniofacial Microsomia and Other Childhood-Onset Disabilities
Source: J Healthc Inform Res. 2023 Sep 18;7(4):480–500. doi: 10.1007/s41666-023-00149-y (PMC10620347; doi:10.1007/s41666-023-00149-y)
Supplement: Supplementary file 2 — (DOCX 17.0 KB) [file 41666_2023_149_MOESM2_ESM.docx]

Appendix 2: Contingency Table and Class Accuracy of Best Performing Models

1. Binary Classification (Strategy vs. Non-Strategy).

|  | **Strategy** | **Non-Strategy** | **Class Accuracy (%)** |
| --- | --- | --- | --- |
| **Strategy** | 293 | 1 | 99.66 |
| **Non-Strategy** | 15 | 6 | 28.57 |

2. Binary Classification of Caregiver Strategies (Extrinsic vs. Intrinsic Strategies).

|  | **Extrinsic** | **Intrinsic** | **Class Accuracy (%)** |
| --- | --- | --- | --- |
| **Extrinsic** | 182 | 13 | 93.33 |
| **Intrinsic** | 30 | 69 | 69.70 |

3. Multinomial Classification of Intrinsic Caregiver Strategies (Sense of Self, Preferences, Activity Competence).

|  | **Sense of Self** | **Preferences** | **Activity Competence** | **Class Accuracy (%)** |
| --- | --- | --- | --- | --- |
| **Sense of Self** | 57 | 1 | 3 | 93.44 |
| **Preferences** | 5 | 11 | 1 | 64.71 |
| **Activity Competence** | 6 | 0 | 15 | 71.43 |

4. Multinomial Classification into the 4 fPRC Participation-Related Constructs and a Non-Strategy Class

|  | **Environment/ Context** | **Sense of Self** | **Preferences** | **Activity Competence** | **Non-Strategy** | **Class Accuracy (%)** |
| --- | --- | --- | --- | --- | --- | --- |
| **Environment/Context** | 185 | 8 | 0 | 2 | 0 | 94.87 |
| **Sense of Self** | 16 | 42 | 1 | 1 | 1 | 68.85 |
| **Preferences** | 9 | 0 | 7 | 0 | 1 | 41.18 |
| **Activity Competence** | 11 | 0 | 0 | 10 | 0 | 47.62 |
| **Non-Strategy** | 18 | 1 | 0 | 0 | 2 | 9.52 |

**Article title**: Natural Language Processing to Classify Caregiver Strategies Supporting Participation Among Children and Youth with Craniofacial Microsomia and Childhood-Onset Disabilities

**Journal name**: Journal of Healthcare Informatics Research.

**Author names and affiliations:** Vera C Kaelin [1, 2], Andrew D Boyd [1, 3], Martha M Werler [4], *Natalie Parde [5, 6], *Mary A Khetani [1, 2, 7, 8]

[1] Rehabilitation Sciences, University of Illinois Chicago, Chicago, USA

[2] Children’s Participation in Environment Research Lab, University of Illinois Chicago, Chicago, USA

[3] Biomedical and Health Information Sciences, University of Illinois Chicago, Chicago, USA

[4] Epidemiology, Boston University, Boston, USA

[5] Computer Science, University of Illinois Chicago, Chicago, USA

[6] Natural Language Processing Laboratory, University of Illinois Chicago, Chicago, USA

[7] CanChild Centre for Childhood Disability Research, McMaster University, Hamilton, CA

[8] Occupational Therapy, University of Illinois Chicago, Chicago, USA

* Co-Senior and Co-Corresponding authors

**Corresponding authors**: Mary A. Khetani, email: [mkhetani@uic.edu](mailto:mkhetani@uic.edu); Natalie Parde, email: [parde@uic.edu](mailto:parde@uic.edu)
